# An experimental examination of the effects of alcohol consumption and exposure to misleading postevent information on remembering a hypothetical rape scenario

**DOI:** 10.1002/acp.3531

**Published:** 2019-03-04

**Authors:** Heather D. Flowe, Joyce E. Humphries, Melanie K. Takarangi, Kasia Zelek, Nilda Karoğlu, Fiona Gabbert, Lorraine Hope

**Affiliations:** ^1^ School of Psychology University of Birmingham Birmingham UK; ^2^ School of Psychology Edge Hill University Ormskirk UK; ^3^ School of Psychology Flinders University Adelaide South Australia Australia; ^4^ School of Neuroscience, Psychology and Behaviour University of Leicester Leicester UK; ^5^ School of Psychology University of Kent Canterbury UK; ^6^ Department of Psychology Goldsmiths, University of London London UK; ^7^ Department of Psychology University of Portsmouth Portsmouth UK

**Keywords:** alcohol, cognitive interview, misinformation effect, rape, self‐administered interview, sexual assault

## Abstract

We experimentally examined the effects of alcohol consumption and exposure to misleading postevent information on memory for a hypothetical interactive rape scenario. We used a 2 beverage (alcohol vs. tonic water) × 2 expectancy (told alcohol vs. told tonic) factorial design. Participants (*N* = 80) were randomly assigned to conditions. They consumed alcohol (mean blood alcohol content = 0.06%) or tonic water before engaging in the scenario. Alcohol expectancy was controlled by telling participants they were consuming alcohol or tonic water alone, irrespective of the actual beverage they were consuming. Approximately a week later, participants were exposed to a misleading postevent narrative and then recalled the scenario and took a recognition test. Participants who were told that they had consumed alcohol rather than tonic reported fewer correct details, but they were no more likely to report incorrect or misleading information. The confidence–accuracy relationship for control and misled items was similar across groups, and there was some evidence that metacognitive discrimination was better for participants who were told that they had consumed alcohol compared with those told they had tonic water. Implications for interviewing rape victims are discussed.

## 1 INTRODUCTION

An estimated 473,000 adults in England and Wales are victims of sexual offenses per year on average (Ministry of Justice, Home Office, & the Office for National Statistics, 2013), and estimates for rape and attempted rape have ranged up to an annual high of 1.27 million persons in the United States (National Research Council, 2014). A recent meta‐analysis found that conviction rates for rapes that were reported to the police have not changed for the past 30 years in Australia, England, Wales, Canada, and the United States—despite legal reforms in these countries to increase prosecution rates, only 12.5% of reports on average result in a conviction (Daly & Bouhours, 2010). One factor that impedes reporting (e.g., Flowe & Maltby, 2018; Wolitzky‐Taylor et al., 2011) and prosecution (e.g., Finch & Munro, 2005) is complainant alcohol intoxication. Victims are typically alcohol intoxicated during rape (Avegno, Mills, & Mills, 2009; Brecklin & Ullman, 2010; Mohler‐Kuo, Dowdall, Koss, & Wechsler, 2004; Palmer, Flowe, Takarangi, & Humphries, 2013; Peterson & Muehlenhard, 2004; Testa, 2002), with some studies reporting victim intoxication rates as high as 70–80% (Government Equalities Office, 2010; Mohler‐Kuo et al., 2004). Testimony from the complainant and defendant is often the primary evidence in rape cases (Lees, 2002), which can be seen as particularly problematic if the case involves alcohol. Psychology and law experts (Kassin, Tubb, Hosch, & Memon, 2001), the police (Evans, Schreiber Compo, & Russano, 2009), and lay people (Benton, Ross, Bradshaw, Thomas, & Bradshaw, 2006; Evans & Schreiber Compo, 2010; Houston, Hope, Memon, & Read, 2013; Lynch, Wasarhaley, Golding, & Simcic, 2013) view testimony as less accurate if it is given by someone who was intoxicated during the crime. Further, even though the police routinely encounter intoxicated witnesses and victims (Crossland, Kneller, & Wilcock, 2018; Evans et al., 2009), there is little police guidance worldwide for how to interview rape complainants who were intoxicated during an incident. According to interview guidance provided by End Violence Against Women International, the only specific guidance, of which we are aware, complainants who were under the influence of alcohol during rape are prone to “filling in the gaps of their memories” (Archambault & Lonsway, 2008). The guidance further asserts: “One of the fundamental challenges to the credibility of sexual assault victims is that many – if not most – make statements to the law enforcement investigator or others that are incomplete, inconsistent, or just plain untrue” (p. 1). Is there empirical evidence to substantiate these views?

Only one study to date has investigated the effects of alcohol on memory for a rape scenario (Flowe, Takarangi, Humphries, & Wright, 2016). Female participants were randomly assigned to consume alcohol or tonic water prior to engaging in an interactive hypothetical rape scenario. Memory for the scenario was examined with a recognition test, which was administered both 24 hr and 4 months later when participants were sober. Women who were alcohol intoxicated (mean breathalysed blood alcohol concentration = 0.08%) answered fewer questions, stating “I don't know” more often, in comparison to their sober counterparts. Accuracy for answered items did not differ depending on alcohol consumption, however. This finding suggests participants tended to answer questions when they felt relatively certain they could provide accurate information. Further research is needed to replicate and extend these findings. Specifically, we need research that measures the accuracy of free recall reports. Moreover, measures of participants' confidence in the likely accuracy of their testimony would also be helpful for examining memory monitoring (e.g., confidence in likely accuracy of information in memory) and control (e.g., volunteering or withholding an answer; responding with “I don't know”) processes.

This paper addresses some of the limitations of previous studies and makes several novel and important contributions. First, we replicated Flowe et al. (2016) using externally valid recall measures to investigate strategic memory encoding and retrieval processes. Participants encoded a hypothetical rape scenario while they were either sober or intoxicated and recalled it 7 days later. We used a balanced placebo design, where half of the participants in each beverage condition were told that they were receiving alcohol, and the other half were told that they were receiving tonic water alone to drink. In the United Kingdom, the policy is for police to obtain an initial account from the victim and then later conduct a much more extensive formal interview (Home Office, 2011). In practice, the police report that they follow this procedure for witnesses and victims who are alcohol intoxicated and sober (Crossland et al., 2018).

In the present study, we interviewed participants about the rape using the cognitive interview (CI) or the self‐administered interview (SAI). U.K. guidelines recommend that all “vulnerable witnesses and victims,” including sexual assault complainants, are interviewed with the CI (Home Office, 2011). The CI is an interview protocol that is widely used in many countries, including the United States and the United Kingdom (Dando & Milne, 2009). The CI provides the interviewee with a series of instructions and mnemonic techniques (e.g., context reinstatement instructions, such as to picture in their mind where they were and what they saw during the crime) to support memory recall. The SAI is a self‐report interviewing tool based on the CI (Gabbert, Hope, & Fisher, 2009; also see Hope, Gabbert, & Fisher, 2011) that U.K. police forces are recommended to use to obtain a first account in certain circumstances (College of Policing [In preparation], n.d.). Such as the CI, the SAI is effective for enhancing memory recall and maintaining memory accuracy over time compared with standard free recall procedures (Gabbert et al., 2009; Gabbert, Hope, Fisher, & Jamieson, 2012; Hope, Gabbert, Fisher, & Jamieson, 2014). In their seminal study introducing the SAI, Gabbert et al. (2009) found that participants who provided an initial account of a mock crime they had witnessed using the SAI remembered as many correct details as participants who were interviewed with the CI; SAI participants also remembered more correct details than control participants (Gabbert et al., 2009). A recent meta‐analysis of 22 research studies found that the SAI has a large effect on increasing correct recall 1 to 3 weeks later compared with when an initial account is not gathered using the SAI (Pfeil, 2018).

Second, we also extended past work by exposing participants to misleading information about the rape. We exposed participants to the misleading information 1 week after the rape scenario, immediately before they were interviewed, to test whether participants who had been alcohol intoxicated compared with sober during the rape were more apt to incorporate misleading information in their memory reports. We delayed the interview because victims are, on average, interviewed 14 days after being raped according to a recent analysis of rape cases that went to trial (Westera, Kebbell, & Milne, 2013). Longer delays possibly increase the likelihood that the complainant is exposed to misleading information about the crime (e.g., via social media or through discussion of the crime with other people) before the interview. Further, people are more likely to include misleading details in their memory reports when misleading information is presented immediately before their memory is tested (Loftus, Miller, & Burns, 1978). Participants who give an initial account captured by the SAI are less likely to later report misleading postevent information and less susceptible to the influence of misleading questions if it is administered soon after the crime and before misleading information is presented (Gabbert et al., 2012; Wang, Paterson, & Kemp, 2014). Similarly, misleading postevent information is less likely to be recalled if the CI occurs prior to misinformation (MI) exposure as opposed to afterwards (e.g., Memon, Zaragoza, Clifford, & Kidd, 2010). Thus, in the present study, we presented participants with MI immediately before the interview to maximize the likelihood of MI reporting to test the effects of alcohol.

Third, we examined the effects of alcohol on the confidence–accuracy relationship. After recalling the event, participants took a recognition test and provided confidence ratings regarding the likely accuracy of their answers, enabling us to examine memory monitoring and control processes. Jurors can find highly confident witnesses persuasive (Douglass, Neuschatz, Imrich, & Wilkinson, 2010); hence, it is important from an applied point of view to test whether highly confident victims are more reliable.

## 2 WHAT IS KNOWN ABOUT ALCOHOL AND MEMORY?

In conventional (non‐eyewitness) memory studies, implicit or automatic memory processes are generally unaffected by acute alcohol intoxication during encoding (Duka, Weissenborn, & Dienes, 2001; Hashtroudi, Parker, DeLisi, Wyatt, & Mutter, 1984; Lister, Gorenstein, Fisher‐Flowers, Weingartner, & Eckhardt, 1991). In contrast, research has found that alcohol intoxication during encoding impairs the recollection of specific episodic memory details, but not feeling of knowing (i.e., familiarity), which may explain why alcohol tends to have a small, if any, effect on recognition accuracy (Bisby, Leitz, Morgan, & Curran, 2009; Mintzer & Griffiths, 2001). Alcohol intoxication during encoding also impairs episodic memory recall in basic memory research (Leitz, Morgan, Bisby, Rendell, & Curran, 2009; Ray & Bates, 2006; Söderlund, Grady, Easdon, & Tulving, 2007) and decreases false memory recall in the Deese Roediger McDermott paradigm, perhaps because of alcohol blocks associative processes (Garfinkel, Dienes, & Duka, 2006; cf. Mintzer & Griffiths, 2001). However, theoretical conclusions reached in these studies may not generalize to applied contexts involving rape. The studies employ verbal learning stimuli (i.e., lists of words; e.g., Garfinkel et al., 2006; Ray & Bates, 2006; Söderlund et al., 2007) or prose (i.e., a news bulletin, which is a subtest of the Rivermead Behavioural Memory Test; Leitz et al., 2009). Memory for rape may be stronger compared with events that are not traumatic, personally involving and complex. Further, past studies (e.g., Leitz et al., 2009) analysed only the number of details correctly recalled, not errors or accuracy rates, confounding recall completeness with recall accuracy. Finally, results from several eyewitness memory studies that have varied alcohol intoxication at encoding are at odds with the basic memory literature. For participants who were intoxicated compared with sober during encoding, recall completeness is lower whereas recall accuracy does not differ (Hagsand, Roos af Hjelmsäter, Granhag, Fahlke, & Söderpalm Gordh, 2013; Harvey, Kneller, & Campbell, 2013; Hildebrand Karlén, Roos af Hjelmsäter, Fahlke, Granhag, & Söderpalm Gordh, 2014; Schreiber Compo et al., 2012; Schreiber Compo et al., 2017; Van Oorsouw & Merckelbach, 2012). A meta‐analysis of these studies (in this Special Issue) found that alcohol intoxication at encoding decreases the number of correct but not incorrect details recalled (Jores, Colloff, Kloft, Smailes, & Flowe, 2019). In the following section, we examine possible explanations for this pattern. We focus on processes that complainants may use to overcome alcohol‐related memory impairments, including strategic attention allocation during encoding and memory reporting strategies when giving testimony.

## 3 ALCOHOL AND REMEMBERING RAPE

### 3.1 Possible strategies at encoding

Attention allocation may affect encoding and memory accuracy, particularly if alcohol has been consumed. Alcohol myopia theory (AMT) proposes that due to alcohol's pharmacological effects, an intoxicated person's attention is allocated to the most immediate and salient cues in the environment (Steele & Josephs, 1990). As a consequence, people allocate less attention to peripheral and weaker cues that conflict with salient ones. AMT has led researchers to predict that people who are alcohol intoxicated compared with sober during encoding will remember peripheral details less accurately, whereas memory accuracy for salient details will be unaffected by alcohol consumption. Evidence for the effect of alcohol on memory for salient versus peripheral details is mixed, however, in the eyewitness memory literature. As predicted by AMT, Schreiber Compo and colleagues found that intoxicated compared with sober participants remember salient details equally well, but they are less likely to recall peripheral details (Schreiber Compo et al., 2011). Other work has found no alcohol‐related differences in remembering salient versus peripheral details, with both intoxicated and sober participants remembering more salient than peripheral details overall (Crossland, Kneller, & Wilcock, 2016, Study 1; Flowe et al., 2016). Still, other research has reported a different pattern of results, with people who were intoxicated compared with sober at encoding reporting just as many peripheral details, but *fewer* salient details (Crossland et al., 2016, Study 2; Van Oorsouw & Merckelbach, 2012). It is not yet altogether clear what accounts for these opposing findings (see Crossland et al., 2016 for a discussion). Nevertheless, all evidence considered, the AMT framework predicts that alcohol intoxication during rape increases selective attention, and thus, victims who were intoxicated compared with sober individuals will recall fewer details about the rape.

Mere knowledge that one has consumed alcohol may also affect attention allocation. Women perceive themselves as particularly vulnerable to sexual assault in situations where they have consumed alcohol (Norris, Nurius, & Dimeff, 1996). According to the *hypervigilance hypothesis*, knowledge that one has consumed alcohol causes women to become more vigilant, to reduce their rape risk (Testa, VanZile‐Tamsen, & Livingston, 2005, as cited in Testa et al., 2006). In line with this hypothesis, in response to a scenario depicting a man making aggressive sexual advances, the highest levels of vigilance were found for female placebo participants, who were misled to believe they consumed alcohol, followed by female participants who had not consumed alcohol, and then participants who had consumed alcohol (Testa et al., 2005, as cited in Testa et al., 2006). Further, in another study, women who were told that they had consumed an alcoholic beverage more accurately remembered a rape scenario compared with participants who were told that they had consumed a placebo (Flowe et al., 2016). Taken together, research suggests attention is strategically allocated during rape depending on alcohol expectancies, and this process affects memory performance. To further test whether hypervigilance leads to greater memory accuracy, we manipulated alcohol expectancy in the present study.

### 3.2 Possible strategies during police interviews

In recalling events, decisions about whether memory output should be suppressed or reported may also lead to improved memory performance in interviews. Research has found that instructing participants to be accurate reduces total output but increases accuracy (Koriat & Goldsmith, 1996). According to the accuracy‐informativeness trade‐off framework, under free report conditions, people answer questions after taking into account the quality of their memory and the costs involved in volunteering versus withholding an answer (Koriat, Goldsmith, & Pansky, 2000). In the eyewitness context, errors of commission may be more consequential than errors of omission (Fisher, Geiselman, & Amador, 1989). This discrepancy may explain why participants in eyewitness memory research trade‐off the completeness of their memory reports to maintain accuracy, reporting information only when they are relatively certain it is accurate (Weber & Brewer, 2008). However, a question that remains is: Does alcohol affect the way in which people make this trade‐off?

In verbal learning research, intoxicated participants respond more conservatively at test than placebo participants (Curran & Hildebrandt, 1999; Maylor, Rabbit, & Kingstone, 1987; Mintzer & Griffiths, 2001, 2002), suggesting they are trying to compensate for expected alcohol‐related memory impairment. There is also basic cognitive research finding that while participants who are intoxicated compared with sober do not differ in judging the likely accuracy of their answers (Evans et al., 2017; Nelson, McSpadden, Fromme, & Marlatt, 1986), they have been found to make less accurate judgements of learning during encoding (Nelson et al., 1998). Findings are somewhat mixed, in the eyewitness/victim alcohol literature. A number of studies have found that participants who were alcohol intoxicated compared with sober when they encoded a crime scenario recall fewer correct details about the scenario, whereas the number of incorrect details they recall does not differ depending on alcohol consumption (e.g., Hagsand et al., 2013; Harvey et al., 2013; Hildebrand Karlén et al., 2014; Schreiber Compo et al., 2012; Schreiber Compo et al., 2017; Van Oorsouw & Merckelbach, 2012). Likewise, Schreiber Compo et al. (2011) found that participants, who thought they had consumed alcohol but in reality had not, were more likely to answer “I don't know” than control and intoxicated participants when remembering an event. Schreiber Compo et al. concluded that a metacognitive control mechanism may operate for placebo participants, causing them to give less complete memory reports to compensate for anticipated effects of alcohol on memory. In line with this idea, Crossland et al. (2016) and Flowe et al. (2016) found that participants who were alcohol intoxicated compared with sober during event encoding were more likely to respond with “I do not know” when their memory for the event was tested while they were sober. In two other studies, however, Schreiber Compo et al. (2012, 2017) found no alcohol‐related differences in the rate of “I do not know” responses. Here, we extend previous work by testing whether alcohol intoxication affects completeness (i.e., the total number of details recalled) but not accuracy when recalling rape, even when people have been exposed to MI.

## 4 SUGGESTIBILITY: ALCOHOL AND THE MI EFFECT

When people remember a crime, their memory report can be less accurate if they have been exposed to misleading information about the event, a finding known as the MI effect. If the original event was weakly encoded, or not encoded at all, the MI effect is more likely (Lindsay & Johnson, 1989; for reviews, see Lindsay, 2008; Mitchell & Johnson, 2009). If alcohol impairs memory, then, we might expect that people who were alcohol intoxicated during the rape will be more apt to incorporate MI in their memory reports.

However, it is difficult to draw predictions from the extant alcohol literature because the methodology used across the few studies that have been conducted varies widely. Van Oorsouw, Merckelbach, and Smeets (2015) did not vary MI exposure but rather subjected their participants to nonleading questioning followed by leading questioning. They found that alcohol intoxication during encoding was associated with increased suggestibility, but only when participants were asked leading follow‐up questions. Gawrylowicz, Ridley, Albery, Barnoth, and Young (2017) found that when people were sober during event encoding and then consumed alcohol just before they received MI, they were *less* likely to incorporate misleading details in their memory reports 24 hr later compared with those who consumed a placebo. Schreiber Compo et al. (2012) found that the likelihood of reporting MI did not differ for sober participants compared with participants who were alcohol intoxicated both during the to‐be‐remembered event and when they were exposed to MI (Schreiber Compo et al., 2012). It is not clear how to generalize these findings to rape. Rape complainants in the United Kingdom are interviewed after a delay, when they are sober, and interviewers are not supposed to ask leading questions (Ministry of Justice, 2011). Under these circumstances, where there is a delay between the crime and the interview, and the victim is exposed to MI during the retention interval, will victim alcohol intoxication during the rape increase susceptibility to the MI effect?

There are theoretical reasons to expect that alcohol increases MI susceptibility. According to source monitoring theory, cues about the source of a memory (such as affective information; perceptual details; day, time, and place information; and the cognitive operations that took place during learning) assist people in differentiating the source of their memories (Johnson, Hashtroudi, & Lindsay, 1993). Retrieval of the original memory trace rather than MI is more likely if people monitor the source of their recollections (e.g., Lindsay & Johnson, 1989; Thomas, Bulevich, & Chan, 2010). Conditions that reduce diagnostic source cue availability (e.g., a relatively long retention interval) lead to poorer metacognitive discrimination and lower accuracy on misled items (e.g., Horry, Colton, & Williamson, 2014). Memory accuracy is lower, and the confidence–accuracy relationship is weaker for misled compared with control items (Bonham & González‐Vallejo, 2009; Cann & Katz, 2005; Loftus, Donders, Hoffman, & Schooler, 1989; Tomes & Katz, 2000), unless participants are warned they have been given MI (Higham, Luna, & Bloomfield, 2011). People who were intoxicated during encoding may have a weaker memory for the original event and fewer source cues available in memory to help them differentiate suggested from original event details. However, whereas alcohol intoxication has not been found to affect the confidence–accuracy relationship in line‐ups (Flowe et al., 2017), the effect of alcohol on recall and metacognitive discrimination accuracy following MI exposure has not been examined.

## 5 CURRENT STUDY

We set out to test several hypotheses. If memory is impaired by having consumed alcohol, then people who were alcohol intoxicated compared with sober during a rape scenario will recall fewer correct and more incorrect details (Hypothesis 1), remember more misleading details (Hypothesis 2), and demonstrate a weaker relationship between confidence and accuracy (Hypothesis 3). On the other hand, participants may expect alcohol to impair their memory and, thus, attempt to compensate for it. If this is correct, then, participants who are led to believe that they had consumed alcohol prior to scenario encoding will give less complete accounts, reporting fewer correct and fewer incorrect details (Hypothesis 4). Further, in line with the hypervigilance account, which proposes that attention is enhanced for those who believe they are intoxicated during the scenario, we hypothesized that women who were told that they had consumed alcohol rather than tonic recall would remember the scenario more accurately and have higher metacognitive discrimination (Hypothesis 5).

## 6 METHOD

### 6.1 Participants

Eighty women aged 18–31 years (*M* = 20.36, *SD* = 2.41 years) who passed a number of prescreenings (described below) participated. They were remunerated (£6 per hour).

### 6.2 Design

We used a 2 beverage (tonic water vs. alcohol) × 2 expectancy (told alcohol vs. told tonic) × 4 information type (consistent, neutral, misled, and control) × 4 scenario man × 4 scenario version mixed design, with information type as the only within participant factor. Participants were randomly assigned to conditions. The dependent variables were measures of free recall, recognition, and confidence.

### 6.3 Materials and procedure

The study received ethical approval from the Psychology Research Ethics Committee at the University of X (location redacted for purpose of blind review). Advertisements for female social drinkers were circulated around the University campus. Potential participants were informed that the study concerned the sexual and dating behaviours of women. Women who responded to the advertisement received further information from the researchers via email. They were informed there would be an initial prescreening and that the study may include discussion of sensitive topics such as rape and sexual assault. The study consisted of eight phases, conducted by female researchers, in which participants took part individually.

#### 6.3.1 Phase 1: Prescreening

Participants completed the prescreening element via on online survey, the link to which was provided via email. The prescreening measures included the Alcohol Use Disorders Identification Test, a 10‐item questionnaire designed to detect hazardous and harmful alcohol consumption (Babor, Higgins‐Biddle, Saunders, & Monteiro, 2001; Saunders, Aasland, Babor, de la Fuente, & Grant, 1993). A general health questionnaire (designed by the researchers) was used to identify any current health problems (i.e., heart or liver disease and psychiatric disorders) and prescription medications that participants were taking. Women were invited to participate in the study if they scored less than 10 on the Alcohol Use Disorders Identification Test, did not have any health‐related problems, and were not taking any prescription medications that interacted with alcohol.

#### 6.3.2 Phase 2: Laboratory screening

Participants were asked not consume any food 4 hr prior and to refrain from drinking alcohol for 24 hr prior to their participation. To confirm eligibility, the experimenter reviewed and verified each participant's responses to the prescreening questionnaires on arrival at the laboratory. Photo identification was checked for proof of age, and a urine‐based pregnancy test was administered to confirm that participants were not pregnant. Weight and height measurements were recorded, and the AlcoHawk Slim Digital Alcohol Breath Tester was used to gauge the percent of alcohol in the participants blood. Like other breath alcohol testers, Alcohawk measures and converts a person's deep‐lung air alcohol level into an estimated blood alcohol content (BAC) measurement. BAC is proportional to the percent of alcohol in a person's breath. Participants were informed that they would not be permitted to leave until their BAC level was less than 0.02%. Participants were also advised they should refrain from driving or operating heavy machinery for the rest of the day. All participants were required to sign a consent form indicating their agreement with these conditions.

#### 6.3.3 Phase 3: Beverage manipulation

Women received either an alcoholic or tonic water beverage, depending on the beverage condition to which they had been assigned. Based on an initial breathalyser reading, all participants were confirmed to have a BAC of 0.00% at the start of the study. We gave women in the alcohol condition three cups containing a mixture of vodka (37.5% proof) and tonic water in a 1:5 ratio. BAC level was 0.06%, which is equivalent to 0.60 g/L or 0.57 g/kg. Attention‐allocation disruptions have been reported for this level of intoxication (Harvey et al., 2013; Lamb & Robertson, 1987) and lower (Clifasefi, Takarangi, & Bergman, 2006). The necessary dosage level required was computed for participants based on their height and weight (see Curtin & Fairchild, 2003). The amount of alcohol administered was 101.86 ml (*SD* = 27.77 ml) on average. Each cup was rimmed with vodka and contained vodka‐soaked limes. Participants did not see their drinks being prepared. They were instructed to consume their beverage at a rate of 1 cup every 5 min (total drinking time of 15 min).

We controlled for alcohol expectancy by following procedures used in previous research (Attwood, Ataya, Benton, Penton‐Voak, & Munafò, 2009). Half of the participants in each beverage condition were told that they were going to consume alcohol, whereas the other half were told they were going to consume tonic water. The cups were clearly labelled with either *Vodka and Tonic* or *Tonic Water* to correspond with the expectancy condition to which they had been assigned.

#### 6.3.4 Phase 4: Sexual assault scenario

Thirty minutes after commencing drinking, participants were breathalysed and then immediately afterwards engaged in the interactive scenario. At this time, the mean BAC was 0.00% (*SD* = 0.00) in the tonic water group and 0.06% (*SD* = 0.02, range: 0.04–0.09%) in the alcohol group.

In total, four scenario locations (bar, party, his house, and her house) were crossed with four different men (i.e., who varied with respect to the description given about the man's hometown, occupation, hobbies, appearance, etc.) to create 16 versions of a dating scenario. Participants were randomly assigned to engage in one version. The scenario was presented via the *participant choice paradigm* (Flowe, Ebbesen, & Putcha‐Bhagavatula, 2007; Flowe, Stewart, Sleath, & Palmer, 2011). This method encourages participants' personal involvement in the scenario, allowing each participant to determine the level of interaction that she has with the man (e.g., whether she accepts a ride home from him and whether she invites him into her house), and how much consensual sexual contact she has with him (e.g., whether she consents when he tries to kiss her). In total, there were 25 scenario stages. After each stage, the participant was asked whether she wanted to continue to interact with the man, or *call it a night* and end the scenario. Each stage of the scenario was presented as written text on a computer screen. The participant also heard, over headphones, the scenario text being read by a female narrator.

In the first stage, introductory information about the setting and general information about the man (his occupation and his music interests and hobbies) was presented alongside his photograph. The photograph was a colour head and shoulder shot of the man. The photo was taken from the Radboud Face Database (Langner et al., 2010). Three photos of young adult Caucasian men were selected from the database, and each participant was randomly assigned to view just one of them to avoid any stimulus specific effects. We have worked extensively with this face database in our past research (Flowe, 2012; Flowe, Klatt, & Colloff, 2014) and selected the top three men with the highest attractiveness ratings (also see Langner et al., 2010). Despite there being four men in terms of the types of biographical details, there were in fact only three pictures of men used; the pictures were randomly presented with the scenario text. The man is described in the first stage of the scenario as acting in a flirtatious manner towards the participant (e.g., complimenting her). Eventually, sexual activity was described as occurring between the participant and the man, and the participant was given the choice to engage in the activity with him or to call it a night. For women choosing to remain in the scenario until the end, consensual sexual activity was depicted. If at any stage, a participant decided to *call it a night*, a legally definable act of rape was described. Once the participant made a choice to continue in the scenario or to *call it a night*, they progressed to the next stage of the scenario (i.e., it was not possible to change the course of action), nor could they return to an earlier stage of the scenario.

Note that regardless of experimental condition, all participants were repeatedly breathalysed at approximately 30‐min intervals throughout the study; this was to prevent women in the tonic water condition inferring the beverage they had consumed. We followed the manufacturer's recommendations in the user manual regarding the operation and care of the breathalyser. Participants in the tonic water condition were required to remain in the laboratory for at least 2 hr following beverage consumption so as to not make it clear to them whether they had consumed alcohol. Participants were never informed of their BAC reading.

#### 6.3.5 Phase 5: MI presentation and event recall

Participants were interviewed 7 days after the completing the scenario. Before their interview, they read, via an online survey using the Qualtrics platform, a written postevent narrative of the Stage 1 (the introductory) part of the scenario on a computer screen. (Recall that all women in the study read Stage 1 of the dating scenario). The instructions that accompanied the narrative stated that the study was investigating police interview procedures in an effort to increase the quality of evidence in cases. Participants were told that they were going to be interviewed about the scenario they had interacted with during Session 1. It was further explained that this was a narrative given by another participant during an interview, and its purpose was to illustrate the next part of the study. Sixteen versions of the postevent summary were used for purposes of counterbalancing the critical items. The narrative contained six *consistent* items that matched the details provided in the original scenario (e.g., the male was 25 years old), six *neutral* items that were congruent with the details of the scenario but that provided non‐specific information (e.g., the male was in his twenties), and six *misleading* items (e.g., the male was 21 years old). The six misleading items deliberately differed from the details of the original scenario but remained consistent with the original syntax (e.g., the male was …) and semantic context of the scenario. After reading the postevent narrative, participants completed an unrelated face matching filler task for approximately 5 min.

Next, participants were randomly assigned to be interviewed with either the Self‐Administered Interview© (Gabbert et al., 2009; Hope et al., 2011) or modified cognitive interview (Holliday et al., 2012). The SAI is based on the CI and consists of five sections that are designed to facilitate recall of a witnessed/encountered event. Section 1 provided participants with background information about the SAI and emphasized the importance of completing all sections in the order presented. Instructions pertaining to the “report everything” mnemonic of the CI were also presented. Participants were instructed to refrain from guessing but to provide the most complete and accurate account possible, including the reporting of partial or trivial event information. In the remaining separate sections, nonleading cues were used to prompt further recall about the male's (perpetrator's) appearance (e.g., hair, complexion, clothing, and distinguishing features), information about the scene/location(s), descriptions of any other persons that were present, and any information about vehicles that were present or involved in the event. The final sections contained a series of questions asking about aspects of the event that participants may have not considered mentioning (e.g., viewing conditions). Participants wrote a total of 643 words on average in the free recall phase (range: 121–2,156, *SD* = 502.41 words) and 1,186 words in the questioning phase (range: 605–3,045, *SD* = 573.93 words). The CI protocol included the rapport building, free recall, questioning, and closure phases and appears in Appendix A. The CI interviews were audio recorded and transcribed for coding purposes. A female experimenter, who was trained in administering the CI and who did not administer the participant's beverage, interviewed the participant. The interview took 16.78 min on average (range: 10.47–28.57, *SD* = 4.58 min). The interview and scenario administration took place in different rooms.

After the interview, the participant completed a multiple choice recognition test as per Flowe et al. (2016) to assess their memory for the original scenario items (neutral and consistent) and acceptance of misled items from the postevent narrative. Participants had the option to answer “I don't know” for each question. The recognition test contained 30 questions about 18 critical items: six consistent items (items from the original scenario), six neutral items (items from the original scenario that were referred to in a non‐specific manner in the postevent narrative), six items on which MI was given in the postevent narrative, and eight no‐information control items (items that were present in the scenario but not mentioned in the postevent summary). Participants provided a confidence rating (0–100% confident, with the scale anchored from “not at all confident” to “completely confident”) regarding the likely accuracy of their answer for each question.

Following the recognition test, participants were asked to indicate whether they considered the encounter with the male to be rape, and whether they would report it as rape to the police. Participants responded to each question using an 11‐point Likert‐type scale, anchored from 1 (*definitely no*) to 11 (*definitely yes*). Participants also indicated whether they thought they had consumed alcohol and rated how intoxicated they felt as a check on our expectancy manipulation as per recommended practice, given that expectancy set manipulations fail to produce effects in the vast majority of studies (see Norris, Mariano, Thomas, Nomenson, & George, 2006, in Testa et al., 2006).

#### 6.3.6 Phase 6: Debrief

Participants were informed that the aim of the study was to investigate whether the degree of intoxication influenced women's interactions with and recall of the events that took place within the scenario. Participants were also remunerated £6 an hour; on average, it took 6 hr in total to complete the study.

### 6.4 Coding and measures

Following the coding system devised in previous research (Holliday, 2003; Wright & Holliday, 2007), reported details were coded as correct or incorrect. A detailed scoring template for each scenario was created. The total number of details for the scenarios ranged from 214 details to 263 details. One point was awarded for each piece of information recalled; a detail was coded only the first time it was mentioned. A detail was coded as *correct* if it was present in the scenario and described correctly (e.g., “red sofa” when this was depicted in the scenario) or as *incorrect* if it was present but described incorrectly (e.g., “black sofa” instead of a “red sofa”). If any of the critical postevent MI items were recalled, these were coded separately as MI intrusions, and if the participant recalled details that were not in the scenario (e.g., “He was driving safely”, or “He broke in through the front door” when the scenario stated he drove and that he broke into her home but did not specify how), these were scored as confabulations. Because we asked participants to imagine themselves in the scenario in order to increase realism, we believe it is appropriate to distinguish between confabulations and incorrect details.

Several dependent variables were computed for each participant for data analysis purposes. For both the free recall and question phases, we calculated for each participant the total numbers of correct, incorrect, and confabulated details, as well as accuracy. We also determined total number of MI intrusions and completeness for each participant. Total correct and total incorrect details were based on the sum of all recalled details that were accurate and inaccurate, respectively. Total confabulations comprised the sum of the number of confabulations recalled. Following past research (e.g., Crossland et al., 2016; Gabbert et al., 2012), accuracy was the number of correct details recalled divided by the sum of the number of correct and incorrect details recalled; confabulated details were not included. Total MI intrusions comprised the sum of the total number of MI details recalled. Completeness (see Holliday et al., 2012) was the proportion of correct details recalled out of all possible details that could have been recalled, depending on where in the scenario participants called it a night.

For the recognition test, we determined for every participant the questions that were relevant for the analysis given the stage at which the participant withdrew from the scenario. If a given question was not relevant (i.e., because the participant had withdrawn from the scenario before the information had been provided), the question was excluded from analysis. For the remaining (relevant) items, the proportion of questions to which the participant had provided answers was calculated, and proportion correct was calculated based on the number of questions to which the participant had provided answers.

### 6.5 Intercoder reliability

Interviews were coded and scored by three independent coders. Intercoder reliability was calculated for total correct and total incorrect details. An independent coder, blind to the experimental conditions, scored 10% of the interview transcripts. According to Kappa's coefficient of agreement, there was a high level of agreement between the coders for total correct details, *r*(6) *=* 0.98, *p* < 0.001, and total incorrect details, *r*(6) = 0.82, *p* < 0.05.

## 7 RESULTS

### 7.1 Preliminary analysis

Eleven women progressed to consensual sexual activity, and because our aim was to examine memory for rape, they were excluded from the analyses that follow, resulting in a final sample size of 69. Further, the manipulation check for the expectancy manipulation indicated that it did not work as intended for 17 women (i.e., nine out of 33 who were told they had been given tonic thought they had been given alcohol; eight out of 36 who were told they had consumed alcohol thought they had been given tonic). Therefore, in line with recommended practice (Norris et al., 2006, in Testa et al., 2006), we also analysed the beverage that women believed they had consumed as the expectancy measure (in the alcohol beverage group [*n* = 37], believed tonic *n* = 10, believed alcohol *n* = 27; in the tonic water beverage group [*n* = 32], believed tonic *n* = 22, believed alcohol *n* = 10). The resulting cell sizes when the data were conditioned on alcohol beliefs were small in some cases, and our statistical power for detecting beverage × alcohol interaction effects was low. Therefore, because the results were largely the same no matter whether we analysed the data using expectancy or alcohol beliefs, we present the results for alcohol expectancy for brevity.

Women who consumed alcohol and/or who were told they had consumed alcohol should feel more intoxicated than their counterparts. As a check on our alcohol manipulations, we analysed feelings of intoxication as a function of study condition using a 2 beverage × 2 alcohol expectancy × 2 interview type analysis of variance (ANOVA). Women reported feeling significantly more intoxicated if they consumed alcohol rather than tonic (*M* = 5.17, *SE* = 0.39, 95% CI [4.39, 5.96] vs. *M* = 0.77, *SE* = 0.46, 95% CI [−0.15, 1.70]), a significant main effect for beverage, *F*(1, 61) = 52.38, *p* < 0.001, *η*_*p*_^2^ = 0.36. No other significant effects were obtained (*F*s < 2.87, *p*s > 0.10). These results indicate that women's self‐reported feelings of intoxication corresponded with the beverage they consumed.

We assessed whether scenario man and scenario version affected participant recall by submitting the total number of correct, incorrect, confabulated, and MI details that participants recalled to multivariate analysis of variance, entering scenario man and scenario version as the between subjects factors. The results indicated no significant effects (*F*s < 1.27, *p*s > 0.24), and so we did not consider scenario man and version further.

The mean number of days between scenario encoding and the interview was 6.98 days (*SD* = 1.40, range: 6 to 17 days). Forgetting may have been more considerable in women who were interviewed after a longer delay than their counterparts, and therefore, it was important to verify that interview delay did not significantly vary across the study factors. A 2 beverage × 2 expectancy × 2 interview type ANOVA conducted on interview delay indicated no significant effects for any of the study factors (*F*s < 2.74, *p*s > 0.11).

The mean number of stages to which women consented was 9.17 (range: 1–22, *SD* = 6.38 stages). Women who withdrew from the scenario at a later stage than others were exposed to more scenario information. Accordingly, it was important to assess whether our manipulations affected scenario withdrawal stage, and hence, the amount of scenario information to which women were exposed. We examined whether the number of scenario stages to which women consented varied in relation to the study factors. The results of a 2 beverage × 2 expectancy × 2 interview type ANOVA conducted on scenario stage indicated no significant effects for any of the study factors (*F*s < 2.86, *p*s > 0.10).

We planned to collect equal numbers of SAI and CI interviews. However, our data collection window was limited to the academic year (which is about 6 months after excluding holiday and examination periods) and to the period of funding (i.e., we had 1 year to complete the study). Midway through data collection, it became apparent that we would not have enough time to run both interview conditions, as participant recruitment was significantly slower than expected owing to the strict participation criteria that we were required to put in place. Therefore, about midway through conducting the study, we stopped running the CI interviews and ran only SAI interviews for the remainder of the study because it was more efficient to do so. This meant that we ran twice as many SAI (*n* = 47, with 23 consuming tonic water, and 24 alcohol) compared with CI (*n* = 22: with 9 consuming tonic water and 13 alcohol) interviews (excluding women who consented to sexual intercourse). In the analyses that follow, we collapsed across interview type because we did not have adequate numbers of participants in each interview condition to include interview type as a factor in the analyses. In thinking about the justifiability of doing this, there are three things to note: First, we did not have any hypotheses about interview type having a differential effect on accuracy depending on beverage and/or expectancy. We included CI and SAI interviews in the first instance to check that the results generalized across different types of interviews. Second, we investigated for all of our dependent variables whether interview type significantly interacted with our main variables of interest (beverage and expectancy), and it did not. For the interested reader, Table 1 presents descriptive statistics for the dependent variables by interview type, and as can be seen, the pattern of findings with respect to beverage and expectancy was largely consistent between interview conditions. There were differences, however, between interview conditions on recall accuracy. A multivariate analysis of variance on proportion correct, and the numbers of correct, incorrect, confabulated, and misinformed details, with interview type as the independent variable indicated a marginally significant effect for interview condition, *F*(5, 63) = 2.17, *p* = 0.07, *η*_*p*_^2^ = 0.15. Follow‐up one‐way ANOVAs on each dependent variable indicated a significant effect of interview type on the number of incorrect details recalled, with a greater number of incorrect details recalled in the CI compared with the SAI condition (*M* = 5.82, *SE* = 0.63, 95% CI [4.56, 7.07] vs. *M* = 3.43, *SE* = 0.43, 95% CI [2.57, 4.28], respectively), *F*(1, 67) = 9.86, *MSE* = 85.70, *p* = 0.003, *η*_*p*_^2^ = 0.13. Further, interview type had a marginally significant effect on the number of correct details, with more correct details recalled in the CI compared with the SAI condition (*M* = 35.50, *SE* = 3.09, 95% CI [29.33, 41.67] vs. *M* = 28.96, *SE* = 2.12, 95% CI [24.73, 33.18], respectively), *F*(1, 67) = 3.05, *p* = 0.08, *MSE* = 641.46, *η*_*p*_^2^ = 0.04. Note, however, as discussed above, there were no trends in the data to suggest that the alcohol variables interacted with interview procedure. Therefore, we collapsed across interview type in carrying out inferential tests. We will return to the issue of interview type, and what further research may be warranted, in Section 8.

**Table 1:** Recall data

	Told tonic		Told alcohol	
	Tonic beverage	Alcohol beverage	Tonic beverage	Alcohol beverage
Total number of correct details				
SAI	30.85 (4.29)	34.82 (4.50)	29.11 (4.10)	23.78 (3.96)
CI	44.50 (5.83)	35.22 (5.89)	35.94 (8.21)	26.59 (5.41)
Total number of incorrect details				
SAI	2.85 (0.86)	4.09 (0.90)	4.45 (0.83)	2.14 (0.80)
CI	5.53 (1.17)	7.54 (1.18)	7.37 (1.65)	4.68 (1.09)
Total number of confabulations				
SAI	2.36 (2.46)	11.20 (2.58)	5.50 (2.36)	3.50 (2.28)
CI	3.67 (3.35)	11.83 (3.38)	13.33 (4.72)	4.72 (3.11)
Total number of MI intrusions				
SAI	0.93 (0.32)	0.89 (0.33)	0.43 (0.30)	1.30 (0.29)
CI	0.76 (0.43)	1.50 (0.44)	1.02 (0.61)	0.54 (0.40)
Proportion accurate details				
SAI	0.89 (0.04)	0.89 (0.04)	0.84 (0.04)	0.80 (0.04)
CI	0.85 (0.05)	0.78 (0.06)	0.80 (0.08)	0.81 (0.05)
Proportion complete				
SAI	0.12 (0.02)	0.14 (0.02)	0.12 (0.02)	0.09 (0.02)
CI	0.18 (0.02)	0.14 (0.02)	0.15 (0.03)	0.11 (0.02)

*Note*. Descriptive statistics for the dependent variables as a function of interview and experimental condition, with data collapsed across free recall and question phases. CI: cognitive interview; SAI: self‐administered interview; MI: misinformation.

### 7.2 Data analysis overview

First, we examined the recall data (i.e., completeness, total correct details, total incorrect details, MI intrusions, total confabulations, and accuracy) as a function of beverage and alcohol expectancy. Whereas the retention interval length between encoding and the interview (hereafter, referred to as delay) was not significantly correlated with completeness (Pearson's *r* = −0.16, *p* = 0.20, two‐tailed), it was significantly correlated with accuracy (Pearson's *r* = −0.46, *p* < 0.001, two‐tailed), suggesting delay should be included as a covariate in the analysis of accuracy. Towards this end, we tested the assumptions of analysis of covariance (ANCOVA; Tabachnick & Fidell, 1983). We constructed scatterplots of delay and the dependent variables, conditioning the plots on the experimental factors. Our inspection of these revealed that the relationship between delay and accuracy was similar across the beverage and alcohol beliefs groups, indicating that the homogeneity of regression slopes assumption was not violated. Further, delay was submitted to a 2 beverage × 2 alcohol expectancy factorial ANOVA, and no effects were significant (*F*s < 1.32, *p*s > 0.25), indicating that delay and the study factors were independent. Second, MI intrusions were analysed with ANCOVA, with beverage and expectancy as the independent variables and delay as the covariate. Delay was significantly correlated with the number of MI intrusions (*r* = 0.34, *p* = 0.006, two‐tailed). The relationship between delay and MI intrusions did not vary as a function of beverage and expectancy, indicating that the homogeneity of regression slopes assumption had not been violated. Third, we present the analyses of the recognition data as a function of the study factors, analysing separately the proportion of questions participants answered and accuracy. Fourth, calibration analyses of confidence and accuracy are presented along with analyses of discrimination accuracy.

### 7.3 Recall completeness

Recall completeness was entered into a 2 beverage × 2 expectancy ANOVA. Descriptive statistics appear in Table 2. Women who expected alcohol gave significantly less complete accounts than those expecting tonic (*M* = 0.11, *SE* = 0.01, 95% CI [0.09, 0.13] vs. *M* = 0.14, *SE* = 0.01, 95% CI [0.12, 0.16], respectively), a significant main effect for expectancy, *F*(1, 65) = 5.19, *p* = 0.026, *η*_*p*_^2^ = 0.07, observed power = 0.612. The main effect for beverage was not significant, *F*(1, 65) = 1.12, *p* > 0.05, *η*_*p*_^2^ = 0.017, observed power = 0.18, nor was the beverage × expectancy interaction effect, *F*(1, 65) = 0.46, *p* > 0.05, *η*_*p*_^2^ = 0.007, observed power = 0.103; post hoc power analysis indicated that the sample size of 1,116 would be needed to achieve 80% power to detect these effects.

**Table 2:** Recall data

	Told tonic		Told alcohol	
	Tonic beverage	Alcohol beverage	Tonic beverage	Alcohol beverage
Proportion				
Completeness	0.14 (0.01)	0.14 (0.01)	0.12 (0.01)	0.10 (0.01)
Accuracy	0.88 (0.03)	0.85 (0.03)	0.83 (0.03)	0.80 (0.03)
Number of details				
Total correct FRP	26.23 (3.02)	25.40 (3.12)	22.62 (3.21)	20.64 (2.79)
Total incorrect FRP	1.71 (0.54)	2.59 (0.56)	2.09 (0.58)	1.05 (0.49)
Total correct QP	9.38 (2.27)	9.50 (2.34)	7.83 (2.41)	4.19 (2.06)
Total incorrect QP	2.04 (0.52)	2.73 (0.54)	2.93 (0.56)	2.03 (0.48)
MI intrusions	1.13 (0.27)	0.87 (0.26)	1.04 (0.23)	0.55 (0.27)
Confabulations	2.81 (1.96)	11.42 (2.03)	7.06 (2.08)	3.94 (1.78)

*Note*. Descriptive statistics for the dependent variables (means [standard error of the mean]) as a function of interview phase and experimental condition, with data collapsed across interview type (self‐administered interview and cognitive interview). FRP: free recall phase; QP, question phase; MI: misinformation.

To summarize, in keeping with Hypothesis 4, women who expected alcohol provided interview accounts that were less complete.

### 7.4 Correct recall

Table 2 presents the recall data as a function of study condition. The number of correct details recalled was entered into a 2 beverage × 2 expectancy × 2 interview phase mixed ANCOVA, with delay as the covariate. Women recalled fewer correct details if they were told they had alcohol compared with tonic (*M* = 13.82, *SE* = 1.21 vs. *M* = 17.63, *SE* = 1.25), a significant main effect for expectancy, *F*(1, 64) = 4.75, *p* = 0.03, *η*_*p*_^2^ = 0.07. There were no other significant effects. The main effect for beverage, *F*(1, 64) = 0.84, *p* > 0.05, *η*_*p*_^2^ = 0.013, observed power = 0.147, and the beverage × expectancy interaction effect, *F*(1, 64) = 0.50, *η*_*p*_^2^ = 0.008, *p* > 0.05, observed power = 0.11, were not significant; a post hoc power analysis indicated that a sample size of 976 would be needed to achieve 80% power to detect these effects.

Thus, contrary to Hypothesis 1, consuming an alcoholic beverage did not decrease the number of correct details recalled. In keeping with Hypothesis 4, alcohol expectancy affected recall, with women recalling fewer correct details if they were told that they had consumed alcohol rather than if they were told tonic water.

### 7.5 Incorrect recall

Table 2 presents the recall data as a function of study condition. The number of incorrect details recalled were entered into a 2 beverage × 2 expectancy × 2 interview phase mixed ANCOVA, with delay as the covariate. The alcohol × expectancy interaction effect was significant, *F*(1, 64) = 5.43, *p* = 0.02, *η*_*p*_^2^ = 0.08, observed power = 0.63. Post hoc *t* tests, with Bonferroni corrections applied (*α* = 0.0167), were carried out to localize the interaction effect. Victims in the real world by and large voluntarily consume alcohol and, thus, are usually aware that they had consumed alcohol. Importantly, among those who were given correct information about the beverage they had consumed, the number of incorrect details recalled did not differ as a function of beverage condition (consumed alcohol and told alcohol *M* = 3.19, *SE* = 0.52 vs. consumed tonic and told tonic *M* = 3.71, *SE* = 0.52), *t*(36) = −0.69, *p* > 0.05. In the alcohol beverage condition, participants who were told they had consumed alcohol as opposed to tonic recalled *fewer* incorrect details (*M* = 3.19, *SE* = 0.52 vs. *M* = 5.25, *SE* = 1.14, respectively), although the difference was not statistically significant, *t*(35) = −1.78, *p* = 0.08, two‐tailed. In the tonic water beverage condition, those who were told they had alcohol rather than tonic water reported *more* incorrect details (*M* = 5.00, *SE* = 0.74 vs. *M* = 3.71, *SE* = 0.52, respectively), although the difference was not statistically significant, *t*(35) = 1.45, *p* = 0.16, two‐tailed. Thus, contrary to Hypothesis 1, alcohol consumption did not increase the number of incorrect details recalled. The pattern of findings was in keeping with Hypothesis 5, but the differences were not statistically significant: Women who had consumed alcohol recalled fewer incorrect details if they were told they had alcohol rather than tonic, whereas those who had consumed tonic water reported more incorrect details if they were told that they had consumed alcohol.

### 7.6 MI intrusions

MI intrusions were submitted to a 2 beverage × 2 expectancy ANCOVA, with delay as the covariate. Descriptive statistics are presented in Table 2. A significant main effect for delay was obtained, *F*(1, 64) = 7.38, *p* = 0.01, *η*_*p*_^2^ = 0.10, with more MI intrusions as delay increased, Pearson's *r* = 0.33. The main effects for beverage, *F*(1, 64) = 2.09, *p* > 0.15, *η*_*p*_^2^ = 0.032, observed power = 0.30, and expectancy, *F*(1, 64) = 0.62, *p* > 0.05, *η*_*p*_^2^ = 0.01, observed power = 0.12, and the beverage × expectancy interaction effect, *F*(1, 64) = 0.20, *η*_*p*_^2^ = 0.008, *p* > 0.05, observed power = 0.07, were not significant; a post hoc power analysis indicated that the sample size of 2,606 would be needed to achieve 80% power to detect these effects.

Thus, contrary to Hypothesis 2, alcohol was not associated with recalling more misleading details.

### 7.7 Confabulations

Confabulations were submitted to a 2 beverage × 2 expectancy ANCOVA, with delay as the covariate. Descriptive statistics are presented in Table 2. A significant beverage × expectancy interaction effect was obtained, *F*(1, 64) = 8.89, *p* = 0.004, *η*_*p*_^2^ = 0.12 observed power = 0.84. There were no other significant effects, *F*s < 1.95, *p*s > 0.16). The interaction effect was examined with *t* tests with Bonferonni corrections applied (*α* = 0.025). Among participants who consumed alcohol, those who were told they had consumed alcohol compared with tonic water recalled significantly *fewer* confabulated details (*M* = 3.90, *SE* = 0.75 vs. *M* = 11.44, *SE* = 3.48, respectively), *t*(35) = −2.40, *p* = 0.02, two‐tailed. Among those who consumed tonic water, those who were told they had consumed alcohol compared with tonic water reported significantly *more* confabulated details (*M* = 7.07, *SE* = 2.40, vs. *M* = 2.82, *SE* = 0.73, respectively), *t*(30), 2.05, *p* = 0.049, two‐tailed. Notably, those who were told accurate information about the beverage they consumed did not significantly differ from each other (told alcohol and consumed alcohol *M* = 3.90, *SE* = 0.75 vs. told tonic and consumed tonic *M* = 2.82, *SE* = 0.73), *t*(36) = 1.01, *p* = 0.32, two‐tailed.

### 7.8 Accuracy

Accuracy scores were submitted to a 2 beverage × 2 expectancy ANCOVA, with delay as the covariate. Descriptive statistics appear in Table 2. A main effect for delay was obtained, *F*(1, 64) = 15.08, *p* < 0.001, *η*_*p*_^2^ = 0.19, observed power = 0.97, with accuracy decreasing as the delay increased, *r* = −0.46. The main effects for beverage, *F*(1, 64) = 0.74, *p* > 0.05, *η*_*p*_^2^ = 0.01, observed power = 0.136, and expectancy, *F*(1, 64) = 2.32, *p* = 0.13, *η*_*p*_^2^ = 0.03, observed power = 0.32), and the beverage × expectancy interaction effect, *F*(1, 64) = 0.15, *η*_*p*_^2^ = 0.0001, *p* > 0.05, observed power = 0.05, were not significant; post hoc power analysis indicated that the sample size of more than 78,000 would be needed to achieve 80% power to detect these effects.

These results are contrary to Hypotheses 1; accuracy did not decrease as a result of having consumed alcohol rather than tonic water. Accuracy was 0.83 for those who consumed alcohol and 0.85 for those who consumed tonic water. The results are also contrary to Hypothesis 5 as accuracy was not higher for women who were told they had alcohol as opposed to tonic water.

All of the recall results taken together, the analyses indicated that alcohol consumption did not decrease recall accuracy, contrary to Hypotheses 1–3. Women who were told that they had alcohol rather than tonic gave less complete accounts, which was in keeping with Hypothesis 4, but their accounts were not more accurate, contrary to Hypothesis 5.

### 7.9 Recognition completeness and accuracy

Table 3 presents descriptive statistics for the recognition data. First, using a MANCOVA, we analysed the proportion of questions on the recognition test that were answered as a function of information type (consistent, misled, neutral, and control), beverage, and expectancy, with delay as the covariate. No significant main effects or interaction effects were found (*F*s < 2.05). A post hoc power analysis indicated that the sample size of more than 1,576 would be needed to achieve 80% power for the global effects analysis. Next, we analysed the proportion of accurate answers given as a function of information (consistent, misled, neutral, and control), beverage, and expectancy, using a MANCOVA, with delay as the covariate. No significant effects were found (*F*s < 1.76, *p*s > 0.14). A post hoc power analysis indicated that the sample size of more than 620 would be needed to achieve 80% power for the global effects analysis.

**Table 3:** Recognition data

Item type	Beverage	Expectancy	Mean	Std. error
Proportion of questions answered				
Consistent	Alcohol	Alcohol	0.78	0.04
	Tonic		0.80	
	Alcohol	Tonic	0.76	0.04
	Tonic		0.81	
Misled	Alcohol	Alcohol	0.70	0.05
	Tonic		0.80	
	Alcohol	Tonic	0.76	0.05
	Tonic		0.82	
Neutral	Alcohol	Alcohol	0.70	0.05
	Tonic		0.71	0.06
	Alcohol	Tonic	0.74	0.06
	Tonic		0.72	
Control	Alcohol	Alcohol	0.71	0.03
	Tonic		0.75	
	Alcohol	Tonic	0.67	0.03
	Tonic		0.75	
Proportion correct for answered questions				
Consistent	Alcohol	Alcohol	0.64	0.05
	Tonic		0.81	
	Alcohol	Tonic	0.69	0.05
	Tonic		0.73	
Misled	Alcohol	Alcohol	0.28	0.05
	Tonic		0.39	
	Alcohol	Tonic	0.28	0.05
	Tonic		0.25	
Neutral	Alcohol	Alcohol	0.48	0.06
	Tonic		0.51	
	Alcohol	Tonic	0.51	0.06
	Tonic		0.61	
Control	Alcohol	Alcohol	0.59	0.04
	Tonic		0.66	
	Alcohol	Tonic	0.62	0.04
	Tonic		0.66	

*Note*. Descriptive statistics for the dependent variables as a function of experimental condition.

Thus, recognition completeness and accuracy were not affected by beverage or expectancy (contrary to Hypotheses 1, 2, 4, and 5).

### 7.10 Confidence–accuracy calibration

**Figure 1:**
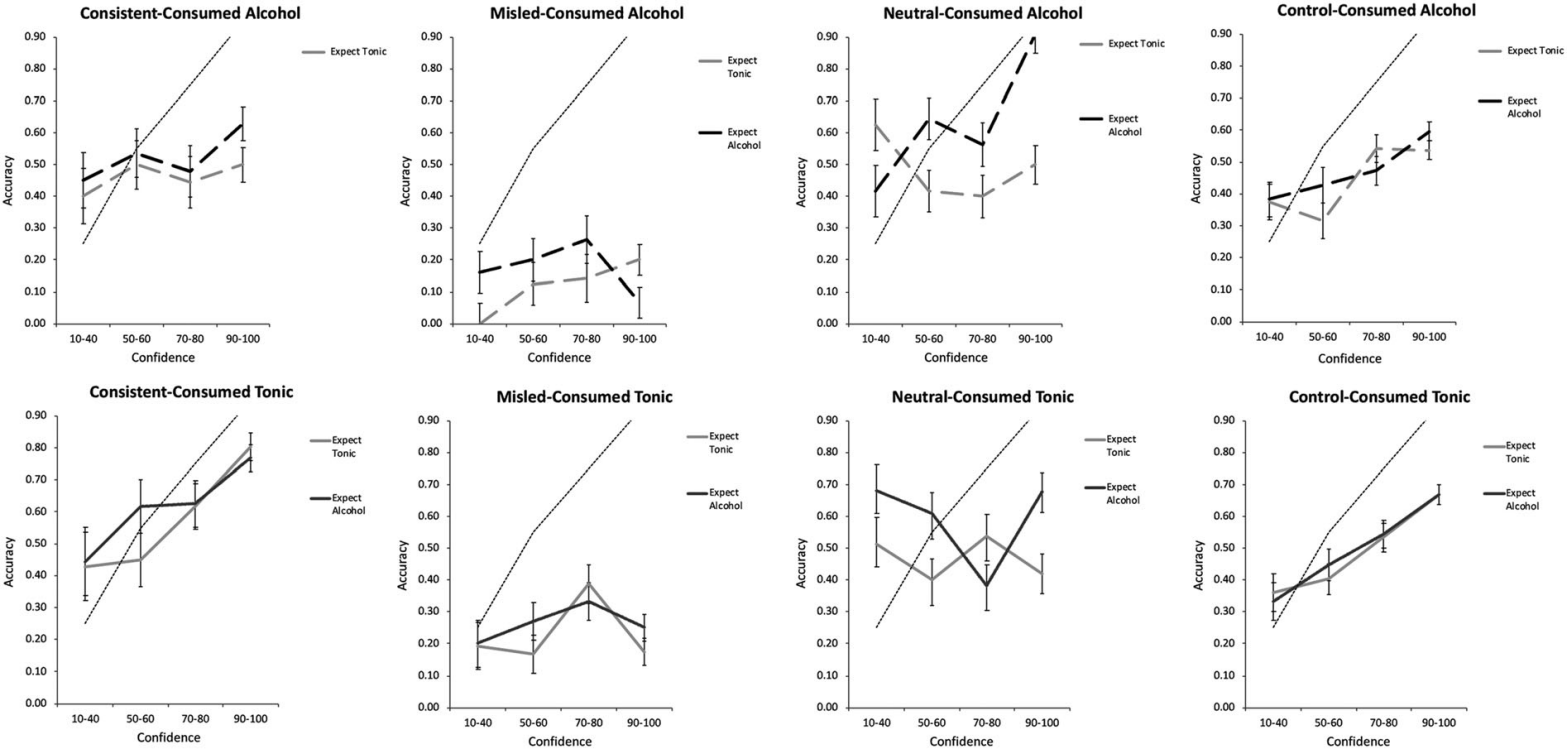
Confidence–accuracy calibration as a function of item type, beverage, and alcohol belief. Error bars represent ±1 standard error of measurement. Dotted grey line represents perfect calibration

Plots of proportion correct as a function of confidence (i.e., calibration curves) by beverage condition and alcohol beliefs are displayed for each item type in Figure 1. Regardless of beverage, accuracy increased with confidence for consistent and control items, whereas the correspondence between confidence and accuracy was relatively weak for neutral and misled items. For all item types, overconfidence tended to increase with confidence level.

We explored whether confidence distinguished correct from incorrect items for sober and intoxicated participants using the adjusted normalized discrimination index (ANDI; see Yaniv, Yates, & Smith, 1991). ANDI ranges from 0 (*no discrimination between correct* vs. *incorrect*) to 1 (*perfect discrimination*), and as can be seen in Table 4, mean ANDI scores tended to be larger for participants who believed they had consumed alcohol compared with tonic water. To examine whether the influence was statistically significant, ANDIs were submitted to a MANCOVA, with beverage and expectancy as the independent variables and delay as a covariate. (Note that ANDI cannot be computed if a participant was always correct, or always incorrect. ANDI could be computed for every item type for 37 participants, and thus, only these participants were entered into the MANCOVA). A significant effect was obtained for expectancy, *F*(4, 29) = 3.23, *p* = 0.03, *η*_*p*_^2^ = 0.21; no other effects were significant, *F*s < 2.09, *p*s > 0.10). A post hoc power analysis indicated that the sample size of 144 would be needed to achieve at least 80% power for the global effects analysis.

**Table 4:** Descriptive statistics for ANDI measure

	ANDI			
	Consistent	Misled	Neutral	Control
Told tonic (*n* = 16)				
Beverage = Alcohol	0.62 (0.19)	0.41 (0.18)	0.24 (0.16)	0.30 (0.12)
Beverage = Tonic	0.70 (0.09)	0.87 (0.09)	0.59 (0.08)	0.37 (0.06)
Told alcohol (*n* = 21)				
Beverage = Alcohol	0.72 (0.09)	0.61 (0.08)	0.71 (0.07)	0.51 (0.06)
Beverage = Tonic	0.63 (0.12)	0.49 (0.12)	0.80 (0.10)	0.35 (0.08)

To follow‐up on the significant result, ANDIs for each item type were separately entered into an ANCOVA, with expectancy as the independent variable and delay as the covariate. For neutral items, discriminability was greater on average for those who believed they had consumed alcohol rather than tonic water, a significant main effect for expectancy, *F*(1, 33) = 10.17, *p* = 0.003, *η*_*p*_^2^ = 0.24. ANDI scores did not significantly vary depending on expectancy for the other item types (*F*'s < 1.33, *p*'s > .25). Thus, alcohol consumption did not appear to weaken the relationship between confidence and accuracy, contrary to Hypothesis 3. Further, there was evidence that women who were told they had consumed alcohol demonstrated better metacognitive accuracy, which is in line with Hypothesis 5.

All of the recognition analyses considered, alcohol consumption and expectancy did not affect recognition performance or the confidence–accuracy relationship, contrary to Hypotheses 1–4. In support of Hypothesis 5, alcohol expectancy did increase metacognitive discrimination, but only for neutral items.

## 8 DISCUSSION

This study investigated the effects of alcohol on memory encoding and retrieval strategies when women remember rape. We tested several hypotheses about whether acute alcohol intoxication during rape impairs memory, including whether people who were alcohol intoxicated compared with sober recall fewer correct and more incorrect details about the rape (Hypothesis 1), recall more misleading postevent details (Hypothesis 2), and demonstrate a weaker confidence–accuracy relationship and reduced discrimination accuracy (Hypothesis 3). No support was found for Hypotheses 1–3. We also tested a number of hypotheses related to alcohol expectancy. Specifically, if participants are told that they are consuming alcohol rather than tonic, they may try to compensate for alcohol‐related memory impairments by providing less complete accounts (Hypothesis 4). Further, as per the hypervigilance account, participants who expect alcohol may encode the scenario better and thereby remember the scenario more accurately and have higher metacognitive discrimination accuracy (Hypothesis 5). We found evidence that participants were attempting to compensate for alcohol's negative effects, in line with Hypothesis 4. We found mixed support for Hypothesis 5. Participants who were told they had alcohol compared with those told tonic water had higher metacognitive discrimination accuracy on the recognition test, but their recall performance did not differ. We will now discuss these findings in relation to the wider literature and their applied implications.

### 8.1 Encoding strategies

The AMT (Steele & Josephs, 1990) framework predicts that alcohol intoxicated victims selectively attend to salient over more peripheral aspects of a crime, thereby causing them to remember less information than victims who were sober. We did not find support for this prediction, as alcohol consumption did not decrease the number of details remembered about the rape. Further, we also tested whether the mere expectation that one has consumed alcohol affects encoding. According to the hypervigilance account, women engage in strategic attention allocation processes to reduce their risk of rape in situations where they are most vulnerable, such as when they are intoxicated (Testa et al., 2006). In keeping with this hypothesis, women who were misled to believe they were alcohol intoxicated as opposed to sober demonstrated increased vigilance in a scenario depicting a man making aggressive sexual advances (Testa, VanZile‐Tamsen, & Livingston, 2005, as cited in Testa et al., 2006). One implication of hypervigilance theory is that alcohol expectancy will increase women's attention during the rape and improve their ability to remember it, all other things being equal. In line with this, women who were told they had been given alcohol rather than tonic water prior to engaging in a rape scenario had higher recognition accuracy when their memory for the scenario was tested (Flowe et al., 2016). However, in the present study, we only partially replicated these results. Women who expected alcohol as opposed to tonic did not recall the rape more accurately. We did find increased discrimination accuracy on the recognition test for neutral items among those who expected alcohol as opposed to tonic water. Possibly, the interview mnemonic techniques enhanced participants' ability to remember the scenario, limiting expectancy effects. The interview procedures may have improved memory retrieval and overshadowed hypervigilance effects on memory, which tend to be small (see Testa et al., 2006).

### 8.2 Memory retrieval strategies

We found that recall reports were less complete for women who were told that they had alcohol compared to those who were told they had tonic. Women provided fewer correct details if they were told they had alcohol as opposed to tonic. There were no alcohol‐related effects on recall errors, however. These results are in line with past research, which has found that the number of correct details is lower for those who were intoxicated compared with sober during encoding, while the number of incorrect details reported does not vary (e.g., Hagsand et al., 2013; Harvey et al., 2013; Hildebrand Karlén et al., 2014; Schreiber Compo et al., 2012; Schreiber Compo et al., 2017; Van Oorsouw & Merckelbach, 2012). The levels of recall accuracy we found—83% of the details recalled were correct for the alcohol beverage group and 85% were correct for the tonic beverage group—are comparable with studies in which participants witnessed crimes other than rape (e.g., Gabbert et al., 2012), suggesting that sexual assault scenarios are not necessarily remembered less well than other types of criminal events.

The recall findings suggest that participants who were told they had alcohol adjust their memory report criterion to a more conservative level to compensate for the negative effects of alcohol on memory. Participants may decrease the completeness of their memory reports in an effort to maintain an acceptable level of accuracy. Shifting the memory report criterion to a more conservative level (i.e., reducing completeness) can decrease the hits more so than the false alarms (see Wickens, 1942). This can explain why the number of correct but not incorrect details reported are lower for participants who consume alcohol in witness studies, as discussed above. On the other hand, the recall strategy people are using to compensate for anticipated alcohol‐related memory impairment does not seem optimal because it causes a decrease in correct but not incorrect recall. Having said this, even though we misled participants, the number of incorrect details they recalled tended to be small, regardless of beverage condition, suggesting floor effects. Few studies have examined the effect of alcohol beliefs on report accuracy. We hope other labs seek to replicate and extend our findings, using methods that increase recall errors overall to test alcohol‐related effects.

### 8.3 The reliability of memory: Confidence and accuracy

We found some evidence that metacognitive discrimination accuracy was higher for participants who believed they had consumed alcohol compared with tonic water alone. Specifically, we found that for items that did not appear in the postevent narrative (i.e., neutral items), metacognitive discrimination accuracy was higher for participants who were told they had alcohol rather than tonic water. Palmer et al. (2013) hypothesized that people are better calibrated to the extent that they take into consideration theory‐based information about factors that can affect their memory accuracy. If it is apparent to people that a given factor weakens memory (e.g., divided attention during encoding), overconfidence (i.e., rating one's memory to have a higher probability of being accurate than it actually is) is reduced. Thus, memory reliability may be improved for intoxicated witnesses and victims if they take into account that they were alcohol intoxicated. Evidence for this already exists in the alcohol literature: On line‐up identification tests, participants who expected and consumed alcohol compared with a placebo were found to demonstrate a stronger confidence–accuracy relationship (Yuille & Tollestrup, 1990). Likewise, women who were alcohol intoxicated compared with sober during a rape scenario tended to be less overconfident in their identifications of the perpetrator from a line‐up, although the difference was not statistically significant (Flowe et al., 2016). If our findings can be replicated, especially at higher intoxication levels, this would hold important implications for theory and practice. Namely, the reliability of memory reports of witnesses and victims may be improved by asking interviewees to take into account factors that may have impacted their accuracy when reporting information. Likewise, the police may elect to harness confidence information when taking statements to gauge the reliability of the information that they are being given. Research with sober witnesses has found that confidence is predictive of accuracy in simulated police interviews (e.g., Roberts & Higham, 2002).

### 8.4 Alcohol and the effects of MI

The present study also tested the effects of exposure to misleading postevent information on recall errors, which is an important extension of previous research on alcohol's effects on remembering rape. Victims of rape often delay rape reporting, which may increase concerns that they may be exposed to erroneous postevent information from the media and other people before they are interviewed. Police interviewers may be concerned that victims who were intoxicated may be particularly prone to filling in the gaps of their memories with details that they learn about the crime from other sources. The present study tested whether women who were alcohol intoxicated during the rape are more apt to incorporate misleading information into their statements. However, there was no evidence that this was the case. Although participants were more likely to report MI with longer delays between scenario presentation and the interview, we did not find that MI intrusions were higher for participants who were alcohol intoxicated compared with sober during encoding.

Our MI findings accord with previous alcohol research that has also measured recall but administered larger doses of alcohol and achieved a higher BAC than we did (Schreiber Compo et al., 2012). Our findings are important because there is little research on the effects of alcohol on MI acceptance, and no research on it in the context of rape. Practically, if questioning includes misleading alternatives, complainants who were intoxicated compared with sober during the crime may be more likely to report erroneous information. In line with this idea, Van Oorsouw et al. (2015) found that alcohol intoxication during encoding was associated with increased suggestibility only when participants were asked leading follow‐up questions. Best practice guidelines (e.g., Ministry of Justice, 2011; Orbach et al., 2000) highlight the importance of asking open‐ended questions in obtaining information, which is also important in investigating sexual offenses (see Kebbell & Westera, 2011; Westera et al., 2013).

### 8.5 Applied implications

How might the results presented here be used to improve investigative interviews with rape complainants who were alcohol intoxicated at the time of the crime? First, the assumption that testimony is more apt to be inaccurate if given by a complainant who was intoxicated compared with sober does not accord with our results. A survey of psychology and law experts found that 90% of experts agreed that alcohol impairs eyewitness performance, 76% thought there was a research basis on the matter, though 95% of experts thought it common sense that memory is impaired by alcohol, and 61% said they would testify (presumably to say that intoxicated witness testimony is less accurate; Kassin et al., 2001). Research conducted since the expert survey was published, however, does not support the blanket assumption that testimony given by intoxicated witnesses and complainants is likely to be incorrect. The results of the present study echo other findings on intoxicated witnesses (Hagsand et al., 2013; Harvey et al., 2013; Hildebrand Karlén et al., 2014; Schreiber Compo et al., 2012; Schreiber Compo et al., 2017; Van Oorsouw & Merckelbach, 2012), with accuracy rates for intoxicated participants no lower than if participants are sober during encoding. We believe that the totality of circumstances ought to be taken into account when evaluating the likely accuracy of testimony (e.g., whether the victim/witness was exposed to MI and the type of interview procedure used). Second, further research is needed to investigate the impact of established nonsuggestive interview protocols on memory reporting in rape. We did not test participants with a standard interview procedure (i.e., one that does not use mnemonic devices), but there is considerable evidence that accuracy is increased and MI effects can be reduced by using the CI (e.g., Köhnken, Milne, Memon, & Bull, 1999; Memon, Meissner, & Fraser, 2010). Nonsuggestive open‐ended interview protocols may be particularly important in gathering information from rape victims. For instance, in real world rape cases, the amount of detail reported by rape complainants in video recorded interviews conducted by specialist interviewers trained in the CI was found to be over 66% greater compared with the testimony given at trial (Westera et al., 2013; also see Westera, Kebbell, & Milne, 2012). Finally, further research is necessary to determine whether rape victims would benefit from the opportunity to write their accounts at the start of an investigation (for discussion of this issue, see Hope et al., 2011), which would minimize the number of times that they have to be interviewed, perhaps thereby reducing secondary trauma.

There are a number of caveats to bear in mind in generalizing results to the legal system. First, our findings may be limited to circumstances in which people are not exposed to leading questioning. Interviewing complainants as early as possible is advisable for preserving and protecting memory accuracy (e.g., Hagsand, Roos af Hjelmsäter, Granhag, Fahlke, & Söderpalm Gordh, 2017). People who have completed an early SAI have been shown to be less susceptible to subsequently presented MI (Gabbert et al., 2012). Our participants waited a week before they were interviewed. Even still, we found low rates of MI recall, and no increased susceptibility to MI recall among those who had consumed alcohol. However, if we had included misleading questions, probed harder during the interview, or increased the delay between the crime and the interview, participants may have been more likely to recall MI, particularly if they had consumed alcohol. Second, MI presentation format could affect the size of the MI effect (Gabbert, Memon, Allan, & Wright, 2004; Wright, Self, & Justice, 2000). Other research using other MI presentation formats (e.g., news reports and misleading questions) would be welcome. Third, similar to other alcohol research (e.g., Conrad, McNamara, & King, 2012; Mintzer & Griffiths, 2001), we estimated BAC using a handheld portable breathalyser; a benchtop model might have yielded more accurate estimates of intoxication. As such, we cannot draw any inferences about how a specific level of intoxication impacts memory. Fourth, the effects of intoxication on memory recall may differ at larger alcohol doses, and police may often encounter intoxicated witnesses with a much higher BAC (see Evans et al., 2009). The overwhelming majority of alcohol eyewitness studies conducted in the lab dose participants to a mean BAC of 0.08% or lower, owing to ethical considerations, which are especially important here because of the additional ethical complexities involved in presenting participants with an interactive rape scenario. Having said that, Van Oorsouw et al. (2015) tested participants in the field who were considerably more intoxicated (mean BAC = 0.16, *SD* = 0.04 in the high dose group) and found that whereas the number of correct details reported was lower for the participants who were the most intoxicated, the number of recall errors did not vary in relation to dose. Fifth, as with other alcohol research (e.g., Bisby et al., 2009), we employed an analogue event. Although participants reported being emotionally affected by rape scenarios administered via the participant choice method (Takarangi, Flowe, & Humphries, 2013), our simulated event is, of course, not akin to experiencing rape. However, we based the scenario on real‐life rape cases and sought to make the experience as interactive as possible with the participant choice methodology (Flowe et al., 2007) in an effort to maximize psychological realism (see Mook, 1983). Sixth, we did not systematically vary whether participants were tested in a sober versus intoxicated state due to resource limitations, and thus, state dependent effects on recall could not be determined. Police investigators have told us that the vast majority of complainants are sober during the CI. Nevertheless, state dependency effects tend to be small and idiosyncratic (Duka et al., 2001; Weissenborn & Duka, 2000), and a recent eyewitness study found no effect on recall (Schreiber Compo et al., 2017). Seventh, participants were interviewed on a single occasion, whereas in actual cases, complainants may be called on to testify on multiple occasions, and additional research on this issue is warranted. Previous research suggests early interview is the best practice in terms of maintaining accuracy over the long term, regardless of whether people are still intoxicated versus sober during the initial interview. Further, previous work has found a similar pattern of findings for intoxicated compared with sober participants who were tested 24 hr and 4 months later (Flowe et al., 2016). Finally, although we did not predict any expectancy × beverage interaction effects, we were not sufficiently powered to detect any such effects. However, post hoc power analyses indicated that data from hundreds more (and in some instances thousands more) participants would be needed, as the effect sizes observed were very small.

In sum, the current findings are of considerable importance for rape victims and legal practitioners. Rape complainant testimony is often dismissed and regarded as inaccurate if the complainant was under the influence of alcohol during the crime. Further, extant guidance for interviewing intoxicated rape complainants states that people who were under the influence of alcohol during the crime will be prone to “filling in the gaps of their memories” (Archambault & Lonsway, 2008). Our study is the first to test this assertion, and our findings indicate this is not the case. In the current research, we replicated Flowe et al. (2016) using recall measures to investigate strategic memory encoding and retrieval processes, finding that alcohol expectancy decreases the completeness of testimony but not its accuracy. Our findings are in line with previous eyewitness studies that have employed different types of criminal scenarios and larger doses of alcohol in some cases (Hagsand et al., 2013; Harvey et al., 2013; Hildebrand Karlén et al., 2014; Schreiber Compo et al., 2012; Schreiber Compo et al., 2017; Van Oorsouw & Merckelbach, 2012). These studies have similarly found that people who were alcohol intoxicated during encoding do not make more recall errors. Importantly, we extended this literature with our finding that memory monitoring and control processes during retrieval were better among those who believed they had consumed alcohol.

## CONFLICT OF INTEREST

The authors have no conflicts of interest in relation to the research reported in this manuscript.

## APPENDIX A MODIFIED COGNITIVE INTERVIEW SCRIPT

1a. Rapport building phase
1b. Explain aims/rules of the interview and transfer control

Transfer of control: “I'd like you to tell me what happened in the scenario you read and heard last week. do not make anything up or guess. It's OK to say you do not know or you are unsure about something. I do not know what happened, so if I say something that's wrong, just tell me I am wrong. And if you do not understand something I say, tell me.

Part of this session will involve you closing your eyes. If that makes you feel uncomfortable, that's fine, I'll just need you to look down and focus on the floor instead. If it's OK with you, I'll record us talking and write down some things, just to help me remember what you say for later on. Do you have any questions?”

2. Free recall phase
2a. Context reinstatement

“OK, so first of all, please close your eyes and picture in your mind the dating scenario you experienced last week. It might help to recall where you were in the scenario, what you visualised, what you were thinking and how you were feeling at the time.” [Pause]

Visualise what happened in your mind and think about the following things:

- Where you were
- What you were doing
- Who you were with
- How you were feeling
- What was happening
- Who was involved
- What you could see and hear in your mind

2b. Report all

“Now I'd like you tell me everything you can remember about the event and the people involved… even things that you think may not be important. Please give me as many details as possible, without leaving anything out, and without guessing about the information. We are only interested in your own memories of the event.” [Pause; wait for response]

*Take notes on this information in the order that they say it happened*.

2c. Change order

“OK so now I'd like to try something different that can help me to remember other information. Please can you tell me about the very last thing that you remember in the scenario” [Pause; wait for response]

“OK thank you. Now tell me what happened *just* before that?” [Pause; wait for response]

*Continue asking this until the interviewee reaches the beginning of the scenario*.

*Only note down any additional information and slot it into the free recall order*.

3. Remember more

“That's great. Can you remember anything more about the scenario?”

4. Questioning phase with mental imagery

“I'm going to ask you a few questions about what you have told me about the scenario”

*Follow‐up on the man/scene/car in the order that they mentioned them. If they did not mention them (e.g., car), ask* “Were there any vehicles involved?” *Use their terminology* (*e.g*., *say* “guy” *if they said guy rather than man*).

e.g., “Please close your eyes again. You mentioned a man earlier. Try and picture that man in your head. Can you tell me anything more about the man?”

[Pause; wait for response]

“Without guessing, can you remember his …

*Only ask for the following details if they have not mentioned them already. Ask line by line*.

- Apparent Age • Height
- Ethnic origin • Weight/Build • Features e.g. Eyes /Ears/Mouth/Nose/etc.
- Hair Colour • Facial Hair • Complexion
- Clothing/Shoes • Accent • Glasses
- Jewellery • Accessories • Scars/Marks/Tattoos”

“Can you remember anything else about the man?”

*The scenarios usually involve two scenes* (*e.g*., *the bar and her house later on*). *Ask about each in turn*.

“You mentioned a [bar] earlier. Try and picture the [bar] scene in your head. Can you tell me anything more about the [bar] scene?”

[Pause; wait for response]

“Again, without guessing, please can you provide a description of the [bar] scene as you remember it? Please include details of where you were, where other people were, the movement of yourself and other people you saw, and also details of any features of the scene.”

*Depending on their response* …

“At the [bar] scene, were other people present who saw what happened?”

“Can you provide a description?”

“Can you remember anything else about the [bar] scene?”

*Now follow up on the second scene*.

“You mentioned that you went back to [your home]. Try and picture [your home] scene in your head. Can you tell me anything more about [your home] scene?”

[Pause; wait for response]

“Again, without guessing, please can you provide a description of [your home] scene as you remember it? Please include details of where you were, where other people were, the movement of yourself and other people you saw, and also details of any features of the scene.”

*Depending on their response* …

“At [your home] scene, were other people present who saw what happened?”

“Can you provide a description?”

“Can you remember anything else about [your home] scene?”

“You mentioned a car … Try and picture the car in your head. Can you tell me anything more about the car?”

[Pause; wait for response]

“Again, without guessing, please can you provide as much detail as you can about the car. For instance:

- Size • Shape • Colour
- Make/Model • Number of Doors • Registration Number
- You mentioned that he drove you back. Can you remember details such as the driving style and speed you were travelling?”

“Can you remember anything else about the car?”

“OK, I'm just going to ask you a few more questions”.

How well in your mind did you see the incident?

How long was the entire scenario? *If they ask about the split scenes, ask for each in turn, and then altogether*.

What were the weather conditions like at the time?

What time of day did the event occur?

Did you view the incident in daylight or artificial light? (Describe if possible).

Are there any particular reasons for remembering the event or the man portrayed in the scenario?

Was anyone involved that you know, or who you have seen before? (If so, where and when?)

5. Closure

“Do you have any questions? Thank you for your help.”
